# Novel cancer stem cell targets during epithelial to mesenchymal transition in PTEN-deficient trastuzumab-resistant breast cancer

**DOI:** 10.18632/oncotarget.9839

**Published:** 2016-06-06

**Authors:** Lichao Sun, Joseph Burnett, Mari Gasparyan, Fangying Xu, Hui Jiang, Chang-Ching Lin, Ila Myers, Hasan Korkaya, Yajing Liu, Jamie Connarn, Huining He, Ning Zhang, Max S. Wicha, Duxin Sun

**Affiliations:** ^1^ Department of Pharmaceutical Sciences, University of Michigan, Ann Arbor, MI, 48109, USA; ^2^ State Key Laboratory of Molecular Oncology, Cancer Hospital, Chinese Academy of Medical Sciences, Peking Union Medical College, Beijing, 100021, China; ^3^ Department of Biostatistics, University of Michigan, Ann Arbor, MI, 48109, USA; ^4^ Department of Biochemistry and Molecular Biology, Georgia Regents University, Augusta, GA, 30912, USA; ^5^ Department of Internal Medicine, University of Michigan, Ann Arbor, MI, 48109, USA; ^6^ College of Pharmacy and Tianjin Cancer Institute and Hospital, National Clinical Research Center of Cancer, Research Center of Basic Medical Sciences, Tianjin Medical University, Tianjin, 300070, China

**Keywords:** trastuzumab resistance, EMT, cancer stem cells, MEOX1, HER2+ breast cancer

## Abstract

Continued use of trastuzumab in PTEN-deficient HER2+ breast cancer induces the epithelial-to-mesenchymal transition (EMT), transforms HER2+ to triple negative breast cancer, and expands breast cancer stem cells (BCSCs). Using cancer cell lines with two distinct states, epithelial and mesenchymal, we identified novel targets during EMT in PTEN-deficient trastuzumab-resistant breast cancer. Differential gene expression and distinct responses to a small molecule in BT474 (HER2+ trastuzumab-sensitive) and the PTEN-deficient trastuzumab-resistant derivative (BT474-PTEN-LTT) provided the selection tools to identify targets during EMT. siRNA knockdown and small molecule inhibition confirmed MEOX1 as one of the critical molecular targets to regulate both BCSCs and mesenchymal-like cell proliferation. MEOX1 was associated with poor survival, lymph node metastasis, and stage of breast cancer patients. These findings suggest that MEOX1 is a clinically relevant novel target in BCSCs and mesenchymal-like cancer cells in PTEN-deficient trastuzumab resistant breast cancer and may serve as target for future drug development.

## INTRODUCTION

Among the four subtypes of breast cancer 15-20% of breast cancers are HER2^+^, which is associated with aggressive clinical course [1]. HER2^+^ breast cancers typically respond well to trastuzumab treatment in early stage diseases [2]. However, in metastatic HER2^+^ breast cancers the majority of patients either demonstrate de novo or acquired trastuzumab resistance after one to two year of treatment [3–5]. Numerous studies have investigated molecular mechanisms associated with trastuzumab resistance including HER2 degradation, overexpression of other tyrosine kinase receptors, and reduced expression of the PTEN tumor suppressor [6, 7]. Inactivation of PTEN has been shown to occur within 40% of HER2+ breast cancer patients and has been correlated with poor prognosis, as well as, adaption of mesenchymal characteristics in vitro[8, 9].

Korkaya et al. have previously demonstrated that trastuzumab treatment in HER2^+^ PTEN deficient cancer cells expands the breast cancer stem cell (BCSC) population [10]. The unique properties of self-renewal and differentiation of the BCSC population is suspected to be responsible for drug resistance [11–13]. Our recent study showed that continued use of trastuzumab in PTEN-deficient HER2+ breast cancer induces the epithelial-mesenchymal transition (EMT) and transform HER2+ to a triple negative like breast cancer, which requires unique treatment options [14]. Liu et.al. proposed that two states of BCSCs, mesenchymal-like BCSCs (CD44^+^CD24^−^) and epithelial-like BCSCs (ALDH+), may exist in equilibrium and can transition between states. mesenchymal-like BCSCs were reported to be primarily quiescent and highly invasive, whereas epithelial-like BCSCs are proliferative, and are localized centrally within hypoxic zones [15]. Conceptually, BCSCs plasticity could alter more differentiated cell morphology (epithelial vs mesenchymal), classical subtype makers, and result in distinct capacities for invasion, metastasis, and drug resistance due to the distinct epigenetic state from which those cells are derived. Identification of dramatic molecular changes following drug induced EMT in lung cancer has proven useful to identify potential new therapies following erlotinib resistance [16, 17]. However, the molecular signatures that are associated with the transition between cell states in PTEN-deficient trastuzumab resistant breast cancer has yet to be completely elucidated.

The purpose of this study is to identify the changes in BCSCs states and reveal novel cancer stem cell targets following the EMT in PTEN-deficient trastuzumab-resistant breast cancer. Our previous study demonstrated that parental HER2+ BT474 and the PTEN-deficient trastuzumab-resistant derivative (BT474-PTEN-LTT) exhibit epithelial and mesenchymal morphology respectively. Here we identify using traditional BCSC markers that while BT474 exhibits no CD44+/CD24- cells and high Aldefluor positive cell percentages the opposite is true following the generation of trastuzumab resistance. RNA-sequencing was employed for global gene expression analysis and to reveal novel targets which could be exploited for therapy following EMT and transition of CSC states. These results independently confirmed the bulk transition to a mesenchymal/basal like phenotype, and alteration in traditional BCSC marker expression.

Interestingly, differential response to the small molecule sulforaphane (SF) was observed in parental BT474 and BT474-PTEN-LTT. SF, a natural compound derived from cruciferous vegetables, has proven effective at abrogating CSCs in a host of cancers. Early evidence suggested it is capable of preventing tumor formation in chemically induced models of carcinogenesis [18]. Further, in breast cancer SF is able to decrease the Aldefluor-positive cell population, suppress mammosphere formation, and prevent secondary tumor formation in vivo [19]. Additional data suggests it can eliminate CSCs and enhance traditional chemotherapeutic efficacy in prostate and pancreatic cancer cell lines [20–22]. Together, these studies support the notion that SF may possess broad therapeutic potential against CSCs, which provides a unique secondary filter to identify potential gene candidates that regulate the mesenchymal state.

Functional gene set enrichment analysis and siRNA knockdown of several candidate genes revealed a set of homeobox transcription factors (specifically MEOX1) as novel potential targets in the PTEN-deficient trastuzumab-resistant breast cancer cells. In tumor biopsies MEOX1 is associated with poor patient survival, lymph node metastasis, and higher cancer stages. Reduced level of MEOX1 by siRNA or small molecule inhibitor could decrease mammosphere and colony formation in vitro, and decreased tumor growth and BCSC frequency in vivo. These findings suggest that unique molecular signatures may regulate mesenchymal and epithelial-like cell states in PTEN-deficient trastuzumab-resistant breast cancer, where MEOX1 is a clinically relevant target to regulating both BCSCs and mesenchymal-like cell proliferation.

## RESULTS

### Transition from epithelial to mesenchymal-like BCSCs and bulk characteristics of parental BT474 and PTEN-deficient trastuzumab-resistant BT474

**Figure 1:**
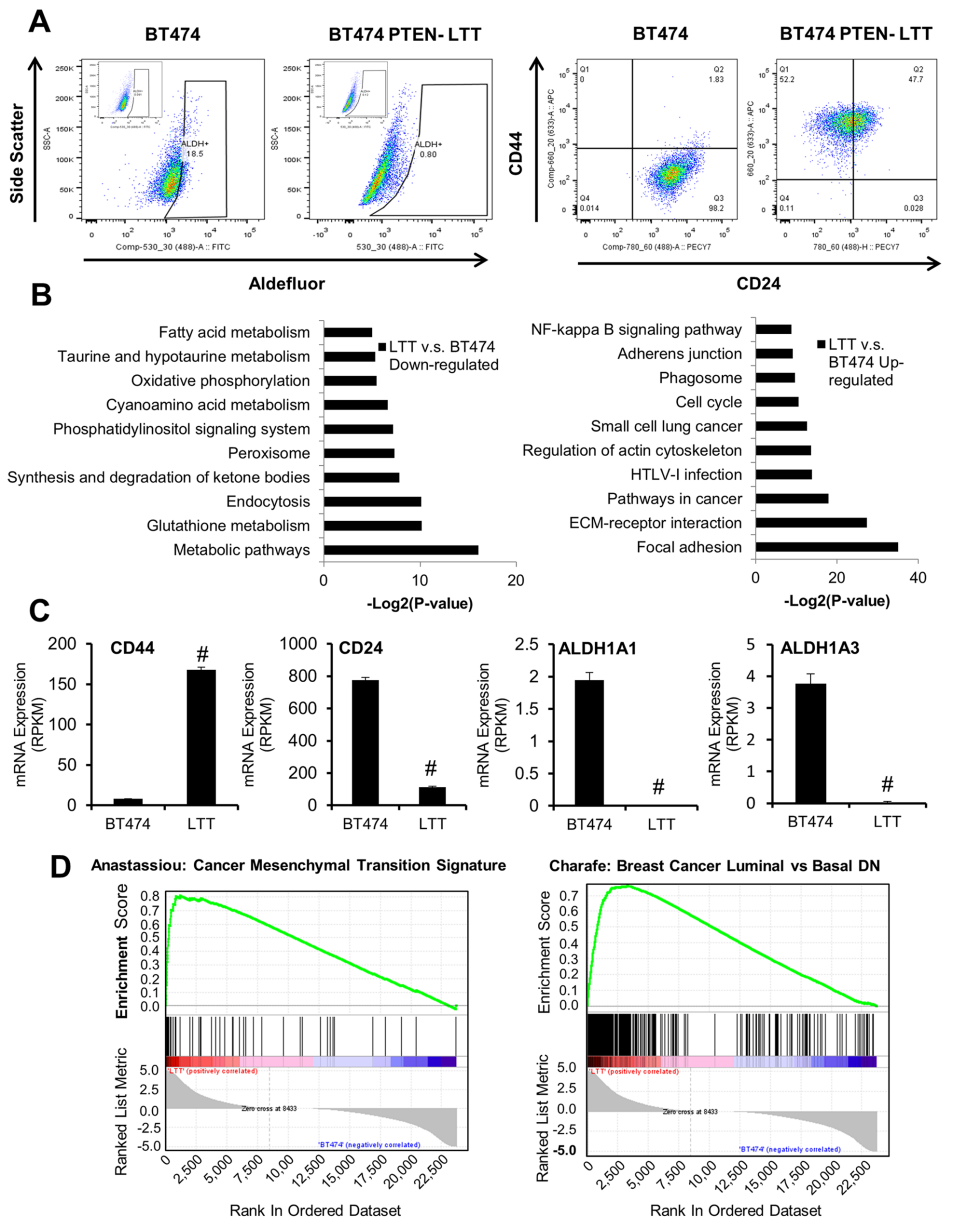
PTEN-deficient trastuzumab-resistant and parental BT474 breast cancer cells exhibit unique BCSC populations and distinct gene expression signatures **A.** Representative flow cytometry analysis of aldefluor positive cells (left) and CD44/CD24 staining (right) in BT474 and BT474 PTEN- LTT cells. Inset, DEAB negative control. **B.** The top ranking gene ontology attributes down-regulated (left) or upregulated (right) following the generation of trastuzumab resistance in BT474 with associated −log2(P-values). **C.** mRNA expression of CD44, CD24, ALDH1A1, and ALDH1A3 in BT474 and BT474 PTEN- LTT cells expressed as reads/kilobase/million mapped reads as determined by RNA sequencing. N=4. Data shown as average ± SD. # p ≤ 0.01. **D.** Gene set enrichment analysis identifying enrichment of upregulated genes in BT474 PTEN- LTT cells (left) and those downregulated (right) pertaining to EMT and molecular subtype.

Our previous studies have shown that continued use of trastuzumab in PTEN-deficient breast cancer cells induced the EMT in bulk cell lines, increased BCSC characteristics, and transformed HER2+ to a triple negative phenotype [10, 14]. However, recent evidence suggests BCSCs may exist in two distinct states, mesenchymal-like (CD44+/CD24-) and epithelial-like (ALDH+), which can interconvert in breast cancer [15]. Therefore, we sought to simultaneously characterize both populations of BCSCs using flow cytometry (Table 1). The HER2 amplified breast cancer cell line BT474 (HER2+ trastuzumab-sensitive) has a distinct population of epithelial-like BCSCs with undetectable mesenchymal-like BCSCs. However, when BT474 cells were genetically manipulated by PTEN inactivation through shRNA knockdown and cultured under long term treatment (LTT) of trastuzumab, the resulted PTEN-deficient trastuzumab-resistant cells (BT474-PTEN-LTT) express primarily mesenchymal-like BCSCs markers and low epithelial-like BCSCs characteristics (Figure 1A).

**Table 1:** Mesenchymal- and epithelial-like BCSCs in BT474 and PTEN-deficient trastuzumab-resistant BT474-PTEN

	Surface markers	PTEN status	Trastuzumab sensitivity	Sulforaphane sensitivity	Mesenchymal-like BCSCs (CD44+CD24-)	Epithelial-like BCSCs (ALDH+)
BT474	HER2+/ER low /PR+	Wild-type	Sensitive	Resistant	0.0-0.3%	29.0-43.0%
BT474 PTEN- LTT	HER2- /ER-/PR-	Low	Resistant	Sensitive	30.7-39.0%	0.7-2.0%

In order to reveal the potential genes involved in the mesenchymal and epithelial phenotypes, we used an RNA-seq approach to compare the transcriptomes of BT474 and BT474 PTEN- LTT cells. While the majority of mRNA expression was consistent between the cell lines (19,811 genes, 83.5%), based on the RVM (Random variance model) algorithm (p-value<0.05, FDR<0.05) and fold change (fold change ≥2 or ≤0.5), we identified 3901 alterations in gene transcription including 2023 up-regulated and 1878 down-regulated genes. Gene Ontology (GO) pathway enrichment analysis was used to categorize the significant genes and related pathways. The results revealed up-regulated genes were significantly enriched for 3 pathways including focal adhesion, ECM-receptor interaction and pathway in cancer progression, whereas down-regulated genes were enriched for primarily metabolic pathways (Figure 1B). In agreement with two types of mesenchymal- and epithelial-like BCSCs status, the levels of ALDH and CD24 were reduced while CD44 was up-regulated in BT474 PTEN- LTT cells compared to parental BT474 cells (Figure 1C).

In order to further explore the global molecular changes in PTEN-deficient and trastuzumab resistant breast cancer cells, gene set enrichment analysis (GSEA) was performed on the differentially expressed genes (Figure 1D). GSEA reveals that the PTEN-deficient and trastuzumab resistant BT474 PTEN- LTT exhibit similar gene expression to cells which have transitioned to a mesenchymal cell type (ES=0.81, p-value<0.01). Further, expression of epithelial markers E-cadherin and EpCAM are specifically within parental BT474 whereas mesenchymal markers N-cadherin and vimentin expression is distinctly within the trastuzumab resistant derivative (Supplementary Figure S1). Interestingly, the genes which were down regulated in BT474-PTEN- LTT were enriched in a data set encompassing genes which are similar in basal breast cancer cells when compared to luminal (NES=0.76, p-value<0.01). These data were in agreement with our recent finding that trastuzumab resistance in PTEN-deficient breast cancer cells induced the conversion to a triple negative phenotype. Taken together, these results suggest that trastuzumab-resistance in PTEN-deficient breast cancer (BT474) induced a transition converting epithelial- to mesenchymal-like BCSCs ultimately converting the HER2+ cell line to basal/triple negative phenotype.

### Distinct response of PTEN-deficient trastuzumab resistant BT474 PTEN- LTT to sulforaphane narrows down gene candidates following EMT of breast cancer cells

**Figure 2:**
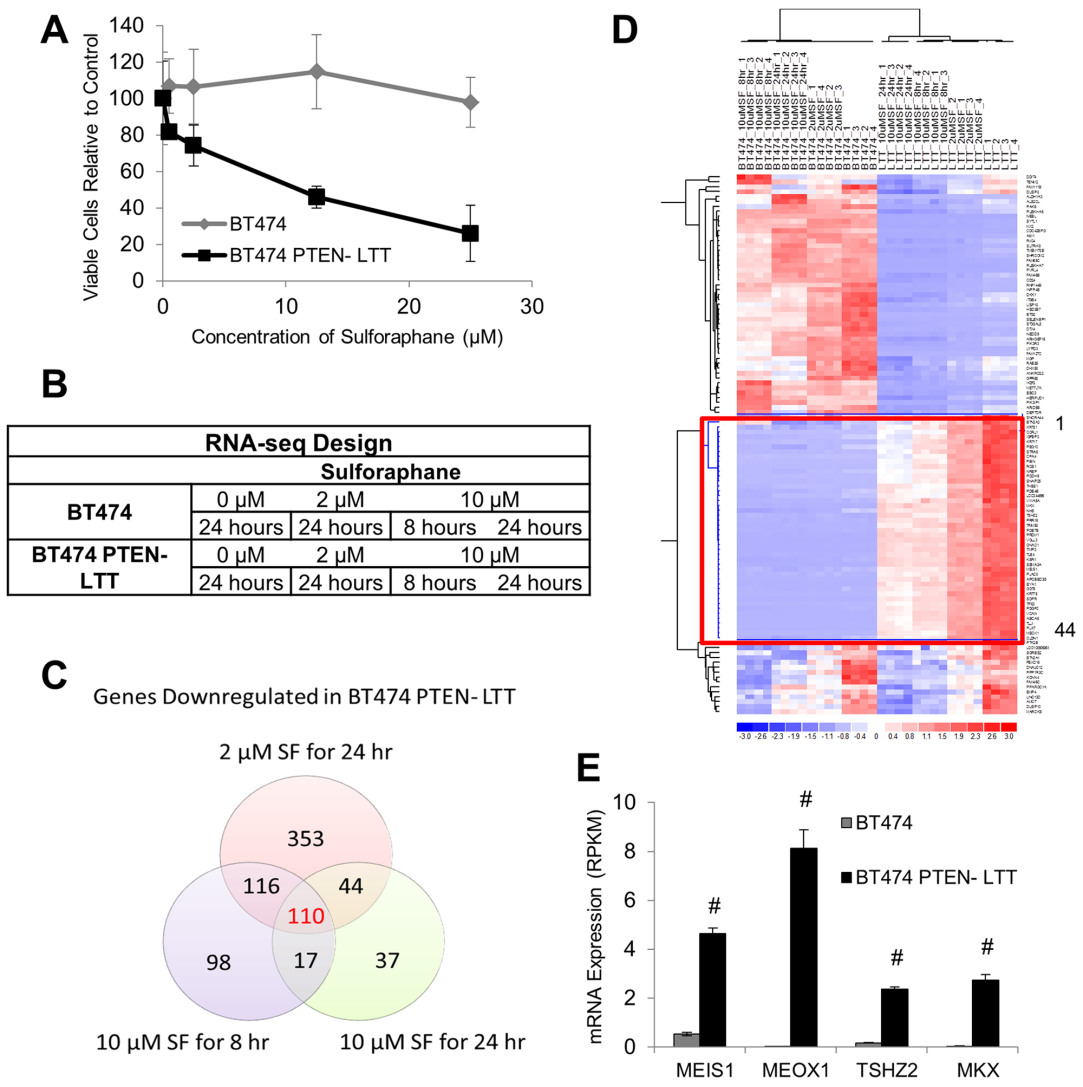
Sulforaphane elicits unique efficacy and gene expression changes in trastuzumab-resistant and parental BT474 cell lines **A.** Cell viability of BT474 and BT474 PTEN- LTT cells following 72 hour treatment with SF as determined by the MTS assay. N=6. **B.** RNA sequencing experimental design for BT4747 and BT474-PTEN- LTT in response to SF to identify time-dependent (10 μM SF 8 hours v.s. 10 μM SF 24 hours) and dose-dependent (2 μM SF 24 hours v.s. 10 μM SF for 24 hours) gene expression changes. N=4. **C.** Venn diagram illustrating number of genes reduced 2-fold with P-value ≤ 0.05 in BT474 PTEN- LTT cells following each treatment. **D.** Heat map illustrating expression changes in BT474 and BT474 PTEN- LTT cells of the 110 genes which are inhibited in a dose and time dependent manner by 2-fold after SF treatment. Red box highlights the 44 genes which were also upregulated in the BT474 PTEN- LTT cell line. **E.** mRNA expression level of the 4 homeobox transcription factors which were functionally enriched from the 44 gene set. N=4. Data shown as average ± SD. # p ≤ 0.01.

Since expression of a great number of genes were altered in BT474 PTEN- LTT and BT474, it is difficult to narrow down novel molecular targets following the EMT. Fortunately, our data showed that trastuzumab resistant BT474 PTEN- LTT responded selectively to sulforaphane (SF) in comparison to parental BT474. Using the MTS cell proliferation assay BT474 PTEN- LTT exhibited high sensitivity to SF (IC50 = 11.5 μM) relative to parental BT474 cells which exhibited no significant response up to 25 μM (Figure 2A). By coupling the treatment of SF with analysis of differentially expressed genes in BT474 and BT474 PTEN- LTT cells, it is feasible to narrow down the molecular targets. Therefore, both parental BT474 and BT474 PTEN- LTT cells were treated with increasing concentrations of SF (2 and 10 μM) to identify dose-dependent changes, and time-dependent gene expression changes at 8 and 24 hours.

Both BT474 and BT474-PTEN- LTT cells were treated with increasing SF concentration and duration, and RNA-seq of total isolated mRNA was performed (Figure 2B). A Venn diagram illustrating the number of genes down regulated by >2-fold in the BT474-PTEN- LTT cells under different treatment conditions reveals that only 110 gene candidates exhibit both time and dose dependent inhibition by SF (Figure 2C). Of these 110 genes only 44 are upregulated by >2-fold in BT474 PTEN- LTT cells relative to the parental BT474 cell line (Figure 2D).

Three methods were used to narrow down fewer candidate genes for further study. (1) Real-time PCR was used to confirm the gene expression differences between BT474 and BT474-PTEN-LTT identified by RNA-seq under identical treatment conditions with SF; (2) siRNA knockdown of the genes with known biological function to assess potential effects on cell proliferation and BCSC characteristics; (3) Functional GSEA using the bioinformatics toolkit DAVID using all identified 44 genes. From these three sets of experiments we selected one functional group, homeobox transcription factors, for additional focus within all candidates (Figure 2E). Among these genes MEOX1 displays the highest mRNA expression in BT474 PTEN- LTT cells and its expression was nearly undetectable in BT474 cells (2989-fold upregulated). Confirmation of RNA-seq results for MEOX1 was performed by real time PCR and similarly demonstrated a 2.5-fold reduction of expression by SF at 24 hours. Therefore, MEOX1 was identified as the top candidate gene for further analysis. A summary of results from functional studies for the top 5 gene candidates can be found in Supplementary Table S1.

### Nuclear MEOX1 expression correlates with patient survival, lymph node metastasis and stage of breast cancers

**Figure 3:**
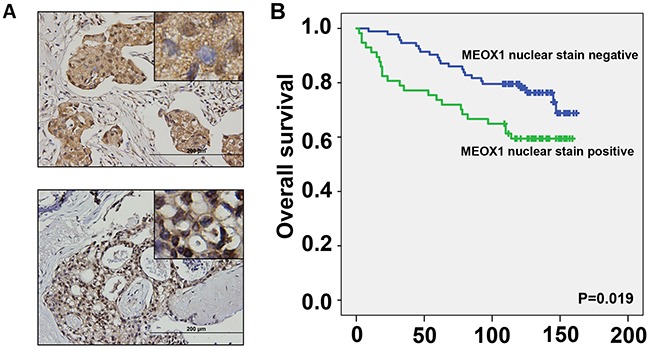
MEOX1 staining in breast cancer tissues correlates with poor patient survival **A.** Representative immunohistochemical (IHC) staining of MEOX1 in patient tumor tissue, top: positive cytoplasmic staining with variable nuclear staining, bottom: strong nuclear staining. Image insets originally obtained using a 40X objective. **B.** Kaplan-Meier survival analysis of the MEOX1 stained TMA when patients were stratified by the presence of nuclear MEOX1 staining. N=150.

In order to investigate the potential clinical implications of MEOX1 in breast cancer patients, we performed immunohistological staining of MEOX1 in a breast cancer tissue microarray (TMA) containing tumor samples and associated survival data (N=150). In this TMA 103 patients survived and 45 patients were deceased, with a median follow-up period of 123.5 months (range, 2-160 months). MEOX1 cytoplasmic staining was positive in 105 primary tumors (Figure 3A, top), whereas nuclear staining was positive in 72 tumor samples (Figure 3A, bottom). Interestingly, the vast majority of the tumor stroma did not show any MEOX1 staining.

Statistical analysis indicated that positive MEOX1 nuclear staining was associated with poor survival of breast cancer patients compared to patients with negative nuclear staining (P =0.019) (Figure 3B). In addition, MEOX1 nuclear staining was significantly correlated with the presence of Lymph node metastasis and cancer stage (P<0.05, Table 2). Further, a Cox regression model was employed to perform multivariate statistical analysis. The analyses revealed that Nuclear MEOX1 expression level (p=0.042), with a Hazard Ratio (HR) of 2.19 and a 95% CI of 1.029-4.669, was an independent prognostic factor in breast cancer patients. No significant correlations were present between cytoplasmic MEOX1 staining and the clinical characteristics noted above.

**Table 2:** Association between MEOX-1 nuclear staining and pathological characteristics of the 150 breast cancer patients

Variable	MEOX-1 staining score		p-value
	0	1	
Age	53.3±13.3	53.7±13.3	0.845
Depth of invasion			0.467
T1+T2	69	61	
T3	6	8	
Lymph node metastasis			**0.011**
N0	35	18	
N1+N2+N3	40	51	
Grade			0.275
I or I-II	22	15	
II, II-III or III	54	56	
Stage			**0.029**
1	9	1	
2	45	41	
3	21	27	

### MEOX1 silencing suppresses the self-renewal of BCSC and mesenchymal-cell proliferation in vitro

**Figure 4:**
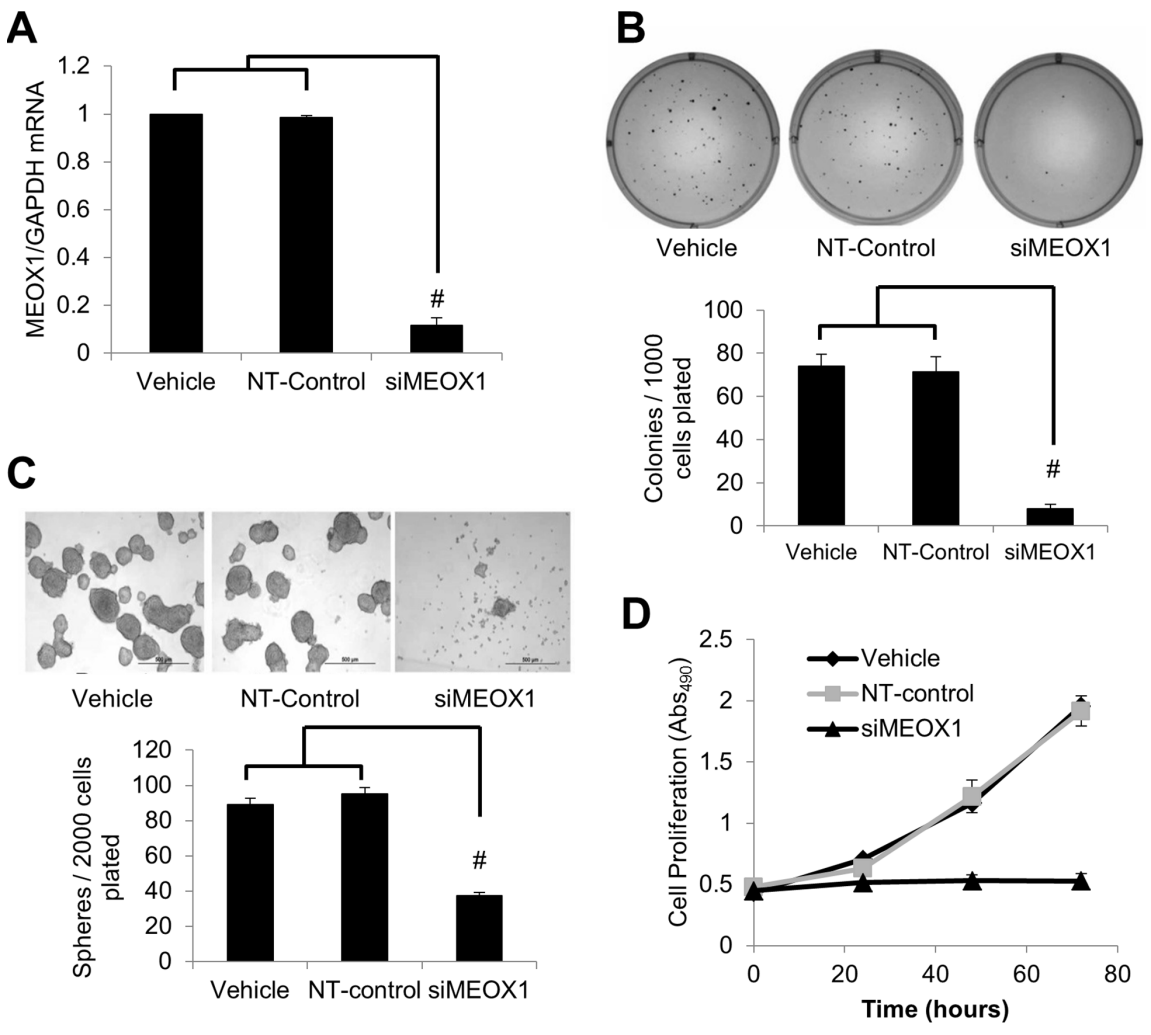
MEOX1 functionally regulates mesenchymal bulk cell proliferation and breast cancer stem cell characteristics in vitro **A.** Real time PCR analysis of MEOX1 mRNA expression relative to GAPDH in transfection reagent (Vehicle), non-targeted siRNA control (NT-control), or siRNA for MEOX1 (siMEOX1) treated BT474 PTEN- LTT cells. **B.** Top, representative images of colonies formed 14 days after siRNA knockdown of MEOX1 in BT474-PTEN-LTT cells in comparison with vehicle and NT-control treatment. Bottom, quantification of number of colonies formed after culture in soft agar. **C.** Top, representative images of mammospheres formed after 7 days following siRNA knockdown of MEOX1 in BT474 PTEN- LTT cells in comparison with vehicle and NT-control. Bottom, quantification of number of mammospheres formed after 7 days of culture in serum free non-adherent conditions. **D.** Proliferation of cells following siRNA knockdown of MEOX1 over the course of 72 hours as determined by MTS proliferation in BT474 PTEN- LTT cells. N=3 in all experiments. Data shown as average ± SD. # p ≤ 0.01.

To elucidate the function of MEOX1 in the mesenchymal BT474 PTEN- LTT cell line siRNA knockdown was employed, reducing expression by 88.3% (Figure 4A). The effect of MEOX1 knockdown on in vitro tumorigenicity was then evaluated using the colony formation assay in soft agar. Colony formation rates following MEOX siRNA treatment were reduced 8.9-fold relative to nontargeted siRNA control in BT474 PTEN- LTT cells over 14 days (Figure 4B). The role of MEOX1 in BCSC self-renewal was also determined using the mammosphere formation assay. Over the course of 7 days, MEOX siRNA treatment led to a 60.5% reduction in the number of mammospheres formed and the size of the average sphere was reduced by 90% (Figure 4C).

Given the importance of proliferation and invasion in cancer progression, we tested whether inhibiting the expression of MEOX1 in BT474 PTEN- LTT cells could affect cell growth by MTS assay and invasion into matrigel. Strikingly, down-regulation of MEOX1 in BT474 PTEN- LTT cells completely inhibited the cell proliferation (Figure 4D). Further, the matrigel invasion assay reveals a 63% reduction in cell invasion following siRNA knockdown (Supplementary Figure S2). Together, these results indicated that MEOX1 might play key roles to regulate BCSCs and mesenchymal cancer cell proliferation in the BT474 PTEN- LTT cells.

### MEOX1 protein expression can be reduced in vitro and in vivo by sulforaphane, which is associated with a decreased frequency of BCSCs and reduction in tumor growth

**Figure 5:**
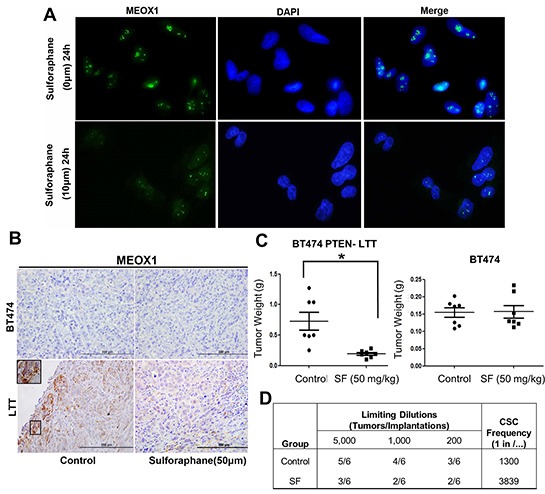
Down-regulation of MEOX1 by sulforaphane in vitro and in vivo is associated with reduced frequency of BCSCs and inhibition of tumor growth in vivo **A.** Representative immunofluorescent staining of MEOX1 in the presence or absence of SF treatment for 24 hrs. MEOX1 (green) resides primarily within the nucleus as evident by overlap with DAPI (Blue) staining. Images obtained originally with 40X objective. **B.** Representative IHC staining of MEOX1 from control or SF treated primary xenografts (BT474 top, BT474 PTEN- LTT bottom) counter stained with hematoxylin and eosin. MEOX1 protein was down-regulated in BT474-LTT-PTEN-LTT by sulforaphane, while BT474 tumor did not express MEOX1. **C.** Left, final tumor mass of orthotopic mouse xenografts (BT474-PTEN-LTT) from mice treated with daily I.P. administration of 0.9% saline or 50 mg/kg SF. Right, final tumor mass of orthotopic mouse xenografts (BT474) treated with SF with the same dose regimen. Data shown as average ± SD. * p ≤ 0.05. **D.** Extreme limiting dilution analysis (ELDA) in secondary mice 8 weeks following implantation of residual cancer cells from primary BT474 PTEN- LTT tumors. Residual cells from SF treated mice exhibit a significantly reduced frequency of tumor initiating cells (P = 0.034).

MEOX1 mRNA is highly expressed in PTEN-deficient trastuzumab resistant BT474 PTEN- LTT, which can be inhibited by SF treatment in vitro. Next, we sought to determine if MEOX1 protein can also be downregulated by SF. MEOX1 protein resides primarily within the nucleus in BT474 PTEN- LTT cells in vitro, as evident by overlap between MEOX1 (Green) and DAPI (Blue) staining (Figure 5A). Treatment with SF (10 μM for 24 hours) led to a reduction in both overall intensity and number of MEOX1 nuclear foci, suggesting SF also reduces MEOX1 protein expression. In order to validate if SF was capable of reducing MEOX1 expression in vivo, immunohistochemistry (IHC) was carried after daily SF treatment (50 mg/kg) in mice bearing orthotopic mouse xenografts of parental BT474 and BT474 PTEN- LTT cell lines. The results demonstrates that SF reduced MEOX1 expression at the protein level in BT474 PTEN- LTT xenografts (Figure 5B, bottom), whereas parental BT474 tumors express no MEOX1 protein regardless of SF treatment (Figure 5B, top).

As demonstrated previously, siRNA knockdown of MEOX1 in BT474 PTEN- LTT cells in vitro resulted in the inhibition of self-renewal of BCSC and bulk cell proliferation. In order to evaluate if the downregulation of MEOX1 was consistent with these observations in vivo, frequency of BCSC and tumor growth in vivo were measured after treatment with sulforaphane (50 mg/kg, I.P). Along with the accompanied MEOX1 downregulation, SF significantly reduced tumor volume by 54% (Figure 5C, left, p-value=0.028) in the BT474-PTEN-LTT xenograft model whereas no inhibitory effect was observed in BT474 xenografts. Further, extreme limiting dilution analysis (ELDA), performed with residual cancer cells from primary mice implanted into secondary recipients, showed that treatment with SF reduced the frequency of BCSCs within these tumors from 1 in 1300 cells to 1 in 3839 cells (Figure 5D, p-value=0.034). Taken together these results provide a proof of concept that inhibition of MEOX1 using a small molecule is a feasible approach to inhibit BCSCs and bulk tumor growth in vivo.

## DISCUSSION

Identification of different breast cancer subtypes has led to significant advances in targeted therapy with unique molecular targets responsible for dramatically different efficacy across subtypes [23, 24]. For HER2+ breast cancers several targeted therapies are currently in use with the front line therapy being trastuzumab. While this antibody has proven extremely useful for early stage HER2+ breast cancer patients, the majority of late stage (metastatic) patients demonstrate de novo resistance or will develop acquired resistance within 1 to 2 years of trastuzumab treatment [3–5]. Several mechanisms have been associated with the generation of trastuzumab resistance including antigen masking, activation of non-canonical HER2 binding partners, or activation of downstream signaling nodes which bypass the requirement for HER2 [25–28]. While second line therapies have been developed for trastuzumab resistant patients, those with distant metastasis will likely succumb to this disease.

Our most recent study has shown that continued use of trastuzumab in PTEN-deficient breast cancer cells induced the EMT, expands breast cancer stem cells (BCSCs)[10], and transform HER2+ to a trastuzumab-resistant triple negative phenotype [14]. These transformed cancer cells show distinct sensitivity to the small molecule sulforaphane, which suggests that different treatment options need to be developed for PTEN-deficient trastuzumab-resistant breast cancer. However, it is still unclear what molecular targets can be exploited for therapy following the observed EMT and apparent subtype switch.

The cancer stem cell (CSC) hypothesis suggests that many types of cancer are sustained by a small population of CSCs, which seem to be responsible for the origin of cancers, tumor recurrence, and drug resistance [13]. In breast cancer, CD44+/CD24- cells or ALDH1+ cells have both been reported to retain CSC characteristics [13, 29]. Recent evidence building on these studies indicates that BCSCs exist in two distinct and dynamic states which may interconvert to form a steady state equilibrium: epithelial-like BCSCs (ALDH+) and mesenchymal-like BCSCs (CD44+CD24-). Mesenchymal-like BCSCs are reported to possess mesenchymal cell type characteristics and are located at the tumor's invasive front. Epithelial-like BCSCs are more proliferative and are located more centrally within tumors [15]. It is possible that the cellular plasticity displayed by BCSCs, coupled with the asymmetric division of either state into more differentiated cells, may ultimately determine the epithelial or mesenchymal characteristics of the bulk cell line however additional studies are to determine this are required.

Accumulating evidence suggests that the induction of EMT and expansion of CSCs may be critical when cancer cells become resistance to trastuzumab. For instance, JIMT-1 cells that exhibit de novo trastuzumab resistance, express relatively high levels of EMT markers SLUG and SNAIL, and are primarily CD44+/CD24-. Conversely, trastuzumab sensitive SKBR3 cells are primarily CD24+ and lack expression of EMT markers [30]. In another report associated with acquired resistance, 3 month culture of SKBR3 cells with trastuzumab generated drug resistance and resulted in expression of EMT inducer TGF-β and downstream target ZEB1 [31]. Further, Lesniak et al. demonstrated rare colonies within the SKBR3 cell line had spontaneously undergone EMT to generated drug resistance and the cells derived from these colonies were primarily CD44+/CD24- with lower HER2 expression [32]. Numerous studies also have indicated that inactivation of PTEN may play important roles in the EMT and trastuzumab resistance. In line with these studies, our work and that by Korkaya et. al. have established that induction of trastuzumab resistance by long term trastuzumab culture in PTEN deficient cells rapidly induces EMT and expands BCSCs [10]. While initially attributed to the activation of the IL-6/STAT3/NF-kB positive feedback loop, it is possible that additional signaling pathways may be critical to proliferation of these cells as well as the self-renewal of the BCSC population.

To further characterize molecular pathways which may play a critical role in the mesenchymal and epithelial states, we investigated the transcriptional landscape using RNA-Seq in cancer cells with primarily epithelial-like BCSCs (BT474, HER2+, trastuzumab sensitive) or mesenchymal-like BCSCs in PTEN-deficient trastuzumab resistant cells (BT474-PTEN-LTT). These experiments identified 3901 differentially-expressed genes between BT4747 and BT474-PTEN-LTT. Utilizing bioinformatics analysis differentially expressed genes were categorized into distinct gene ontology classifications. From them, most up-regulated genes were significantly enriched for 3 pathways including focal adhesion, ECM-receptor interaction and pathway in cancer progression. These pathways in cancer progression included cytokine/NF-kB, HIF-1α, and WNT signaling, all of which are known to play a role in regulating BCSCs and have been associated with EMT [19, 33–35].

In a recent work by Martin-Castillo et.al. the authors propose that the intrinsic molecular subtypes of a breast cancer, coupled with the distribution of epithelial and mesenchymal-like CSC states, can be used to predict the response to trastuzumab in the wide spectrum of clinically diagnosed HER2+ malignancies. Within this framework it is suggested that adaption of mesenchymal traits (EMT) and presence of CD44+/24- cells would coincide with the generation of acquired trastuzumab resistance [36]. Our unbiased GSEA demonstrates the trastuzumab resistant BT474 PTEN- LTT cells appear to have undergone the EMT and adopted a basal like breast cancer phenotype. Furthermore, the RNA-seq data confirmed ALDH1 and CD24 were reduced, and CD44 was up-regulated in BT474-PTEN-LTT cells compared to BT474. These results indicate that continued use of trastuzumab in PTEN-deficient breast cancer cells leads to a BCSC transition from an epithelial- to mesenchymal-like state and support the model proposed by Martin-Castillo et. al.

Due to the number of up regulated genes following the induction of trastuzumab resistance, a knowledge based approach to determining druggable targets is difficult. However, the differential sensitivity of BT474 PTEN- LTT and parental BT474 cells to SF has provided another filter to narrow down the potential target genes. We and other groups have previously shown that SF is able to preferentially inhibit the CSC population in cancer cell lines and suppress secondary tumor formation in vivo [19–21, 37]. The efficacy of SF toward inhibiting CSCs and ability to elicit distinct effects in BT474 and BT474 PTEN- LTT cells make it a valuable small molecule to interrogate unique molecular targets in CSCs.

Since only PTEN-deficient trastuzumab resistant BT474 PTEN- cells are sensitive to SF, we chose to focus on genes that were decreased in both a dose and time dependent manner by SF. This filtering scheme allowed us to narrow our search to only 44 genes. Experimental confirmation (PCR and siRNA knockdown) and functional classification analysis identified four homeobox transcription factors as potential targets. Among them MEOX1 displays the highest expression and fold-change difference between parental BT4747 and BT474-PTEN- LTT cell lines and was therefore selected as the top candidate gene for further study. It is worth noting that many other genes may also be involved in EMT and MET, which require further investigation; but MEOX1 was one of the most specifically upregulated genes within mesenchymal cancer cells in this study.

The MEOX1 (previously MOX1) homeobox transcriptional factor represents a critical mediator of normal somite formation in a developing embryo, a process which requires both the EMT and MET process [38–41]. In patients, homozygous truncation mutations in the MEOX1 gene cause an autosomal-recessive form of Klippel-Feil Syndrome, a disease characterized by fusion of cervical vertebrae [42, 43]. Recent evidence in zebra fish demonstrates MEOX1+ cells also regulate normal hematopoietic stem cell formation in a cytokine dependent manner [44]. Further, MEOX1 has been shown to mediate Hedgehog signaling by regulating Gli1/2 expression during cardiomyogenesis [45].

While the role of MEOX-1 in cancer has largely been unexplored, one study by Thiaville et. al. suggests that this transcription factor partially mediates PBX1 signaling in ovarian cancers [46]. PBX1 has been shown to be a downstream target of NOTCH signaling in breast cancer, and NOTCH itself is a known regulator of CSCs [47, 48]. Initial evidence from our laboratory indicates knockdown of PTEN via shRNA is capable of increasing, while HER2 overexpression suppresses, MEOX1 expression (MCF7 and SUM159, data not shown). Together, this data suggests that there may be direct regulation of MEOX1 by the HER2 signaling cascade beyond the context of trastuzumab resistant HER2+ breast cancers.

In order to study the potential clinical application of MEOX1, TMA staining of a diverse range of breast tumor samples was carried out. Interestingly, cytoplasmic expression of MEOX1 was present in the majority of tumors but limited particularly to cancer cells. Within the cancer cells nuclear protein localization of MEOX1 was correlated with poorer overall survival, an advanced tumor stage, and the presence of lymph node metastasis.

To elucidate the function of MEOX1, its expression was reduced by siRNA knockdown in the PTEN-deficient trastuzumab resistant cell line, which resulted in a significant reduction of BCSC self-renewal and proliferation of bulk BT474 PTEN- LTT cells. Further, knockdown of MEOX1 by siRNA was capable of reducing proliferation of the basal like SUM149PT cell line by 45% (data not shown). Downregulation of MEOX1 by SF in the BT474 PTEN- LTT cell line in vitro and in an orthotopic mouse xenograft model in vivo was able to effectively inhibit bulk tumor volume and reduce frequency of BCSCs, as evident by ELDA in secondary mice. These results demonstrate the feasibility of regulating MEOX1 using small molecule inhibition in vivo. However, additional studies may be necessary to identify the upstream and downstream targets of MEOX1 and its role during the epithelial and mesenchymal transitions.

In summary, these data showed that continued use of trastuzumab in PTEN-deficient breast cancer induces a transition between mesenchymal- and epithelial-like BCSC states and transforms luminal HER2+ cells to a basal like phenotype. Using these cell lines we identify novel cancer stem cell targets in PTEN-deficient trastuzumab-resistant breast cancers. MEOX1 was identified as a clinically relevant molecular target regulating both BCSCs and mesenchymal bulk cell line proliferation. These results may provide a framework for future development of novel therapeutics for the treatment of PTEN-deficient and trastuzumab resistant breast cancers.

## MATERIALS AND METHODS

### Cell lines and reagents

BT474 was cultured in DMEM supplemented with 10% fetal bovine serum and 1% antibiotic-antimycotic under a 5% CO2 environment. BT474 PTEN- LTT cells were generated by lentiviral infection to introduce PTEN shRNA, followed by single cell colony formation to establish a genetically identical cell line. Long term treatment with trastuzumab was carried out for greater than 3 months as previous described [10, 49], and were otherwise maintained in the same media as the parental cell line. Sulforaphane was obtained from LKT Laboratories. Polyclonal antibodies against MEOX1 were purchased from Abcam (ab75895).

### RNA-seq data analysis

Total RNA was extracted using RNeasy Mini kit (Qiagen) and mRNA library was prepare for RNA-seq (poly A selection based) using Illumina TruSeq technology (Illumina). The generated libraries were sequenced on Illumina Hi-Seq 2000 with 50 cycle single ended reads. RNA-Seq reads were aligned to annotated RefSeq transcripts using Bowtie. Only uniquely mapped reads were used for further analysis. Gene expression is expressed as reads/kilobase/million mapped reads (RPKM) and differences in gene expression were estimated using rSeq. Gene set enrichment analysis was performed using the GSEA Java desktop software application (Broad Institute). Finally, gene functional classification was performed using the DAVID Bioinformatics Resources v6.7.

### Knockdown by siRNA

Small interfering RNAs for gene MEOX1 were purchased from Qiagen (validated FlexiTube siRNA, SI00630266). Transfection of BT474 PTEN- LTT cells was carried out using Lipofectamine® RNAiMAX vehicle according to the manufacturer's instruction following optimization. As a negative control, a non-targeting sequence siRNA was utilized (Qiagen, catalog number 1027281). Knockdown at mRNA level was confirmed by isolating total RNA (RNeasy Mini kit, Quigen) and performing real-time quantitative RT-PCR in triplicate. Real-time PCR was carried out on an ABI PRISM 7900HT sequence detection system (Applied Biosystems).

### MTS cell proliferation assay

Cell lines were seeded at a density of 3,000 cells per well in 96-well plates and allowed to adhere overnight. Cells were then incubated with SF in increasing concentrations for a period of 48 hours. Proliferation was determined by MTS assay according to manufacturer's instruction by measuring the absorbance at 490 nm on a Synergy 2 plate reader (Biotek).

### Advanced tumor model

The use of vertebrate animals in this study was conducted in accordance with a standard animal protocol approved by the University Committee on the Use and Care of Animals at the University of Michigan. 5 week old female non-obese diabetic/severe combined immunodeficient (NOD/SCID) mice were obtained from Jackson Laboratory. BT474 or BT474 PTEN- LTT cells (500,000) mixed with Matrigel (BD Biosciences) were injected to the mammary fat pads of NOD/SCID mice. Tumors were measured by caliper and the volume was calculated using V = 1/2 (width^2^ × length). When tumor volume reached approximately 40 mm^3^, the mice were randomly separated into two groups, once receiving daily i.p. injected with 0.9% saline solution and the other receiving 50 mg/kg sulforaphane daily. Final tumor mass was measured on an analytical balance after primary control treated tumor volume reached an average of 500 mm^3^.

### Secondary reimplantation

Isolated primary tumors were mechanically dissociated by mincing with scalpels and suspended in Media 199 and single cell suspensions generated by incubation with collagenase and hyaluronidase (Stem Cell Technologies). Human tumor cells with DsRed label were then isolated using FACS on a SY3200 (Sony Biotechnology) flow cytometer. Secondary female, 5 week old, NOD/SCID mice were inoculated with 5,000, 1,000, or 200 cells for BT474 PTEN- LTT xenografts as described above. Tumor formation rate in secondary mice was assessed 8 weeks following implanting cells by direct palpitation and used to assess cancer stem cell frequency by extreme limiting dilution analysis.

### Colony formation assay

Colony formation was carried out in six-well plates layered with 1.5 mL of 0.5% agar (Difco Agar Noble) in Dulbecco's modified Eagle's medium supplemented with 10% fetal bovine serum and 1% penicillin-streptomycin. Subsequently, 1000 cells mixed with 0.35% agar and allowed to set in each well of the six-well plates in order to form the upper gel. After 2 weeks, pictures of colonies were taken using a digital camera after staining with 0.005% crystal violet. Each treatment was performed in triplicate.

### Immunofluorescence

Cells were seated in glass chambers slides and allowed to adhere overnight. After rinsing with PBS, cells were fixed in PBS containing 4% paraformaldehyde for 30 minutes and permeabilized with 0.1% Triton X-100 (Roche Diagnostics) for 10 minutes at 4°C. Cells were then incubated with MEOX1 (1:200) primary antibodies, followed by FITC-conjugated anti-rabbit IgG (1:300) secondary antibodies. Samples were then mounted and visualized using fluorescent microscopy with a Nikon Eclipse TE2000-S microscope and the photos were acquired with MetaMorph 7.6.0.0.

### Mammosphere formation assay

Mammosphere culture was done as previously described in a serum-free mammary epithelium basal medium (Lonza, Inc.) supplemented with B27 (Invitrogen), 1% antibiotic-antimycotic, 5 μg/mL insulin, 1 μg/mL hydrocortisone, 4 μg/mL gentamicin, 20 ng/mL EGF (Sigma-Aldrich), 20 ng/mL basic fibroblast growth factor (Sigma-Aldrich), and 1:25,000,000 β-mercaptoethanol (Sigma-Aldrich). Single cells prepared from mechanical and enzymatic dissociation were plated in six-well ultralow attachment plates (Corning) at a density of 500 cells/ml. After 7 days of culture, the number of mammospheres was counted on a Nikon Eclipse TE2000-S microscope and the photos were acquired with MetaMorph 7.6.0.0.

### Immunohistochemistry and tissue microarray staining

Immunohistochemical analysis was performed on isolated tumors from mouse xenografts and patients tissues on a tissue micro array (TMA) obtained from US Biomax (HBre-Duc150Sur-01). Standard Envision method was performed. Briefly, after removal of paraffin in xylene and rehydration in graded alcohols, heated antigen retrieval was performed in citrate buffer (10 mmol/L pH 6.0) by microwave heating for 20 min. Endogenous peroxidase activity was prevented by incubation in 0.3% hydrogen peroxide for 10 min. Nonspecific binding was blocked by incubation in 10% normal animal serum for 30 min. Sections were incubated at 4°C for 24 h with MEOX1 (Abcam, ab23279) antibody, diluted 1:100. For blank controls, the primary antibody was replaced with PBS solution (100 mM, PH 7.4). The percentages of MEOX1 positive cells were scored using the following scale: 0= no staining or less than 5%; 1=5-25%; 2=26-50%; 3=51-75%; 4=more than 75%. The intensity of staining was evaluated as 1 (low), 2 (moderate), and 3 (strong). MEOX1 in nucleus was defined as negative (combined score from 0 to 6) or positive (combined score from 8 to 12).

### Statistics

For IHC staining univariate survival analyses were performed and survival curves were drawn using Kaplan-Meier method. The differences between curves were tested by the log-rank test. Comparison of the variables was made by χ2 test. Correlation coefficient was calculated by spearman rank correlation test. For additional studies comparing parental to the drug resistant cell line, siRNA, or sulforaphane treatment statistical differences were determined using two-tailed Student's t test or ANOVA followed by Dunnett post hoc analysis. Data are presented as mean ± SD (n ≥ 3).

## SUPPLEMENTARY FIGURES AND TABLE


