# Supplementary material for: Novel cancer stem cell targets during epithelial to mesenchymal transition in PTEN-deficient trastuzumab-resistant breast cancer
Source: Oncotarget. 2016 Jun 6;7(32):51408–22. doi: 10.18632/oncotarget.9839 (PMC5239484; doi:10.18632/oncotarget.9839)
Supplement: Supplementary file 1 [file oncotarget-07-51408-s001.pdf]

Novel cancer stem cell targets during epithelial to mesenchymal transition in PTEN-deficient trastuzumab-resistant breast cancer

SUPPLEMENTARY FIGURES AND TABLE

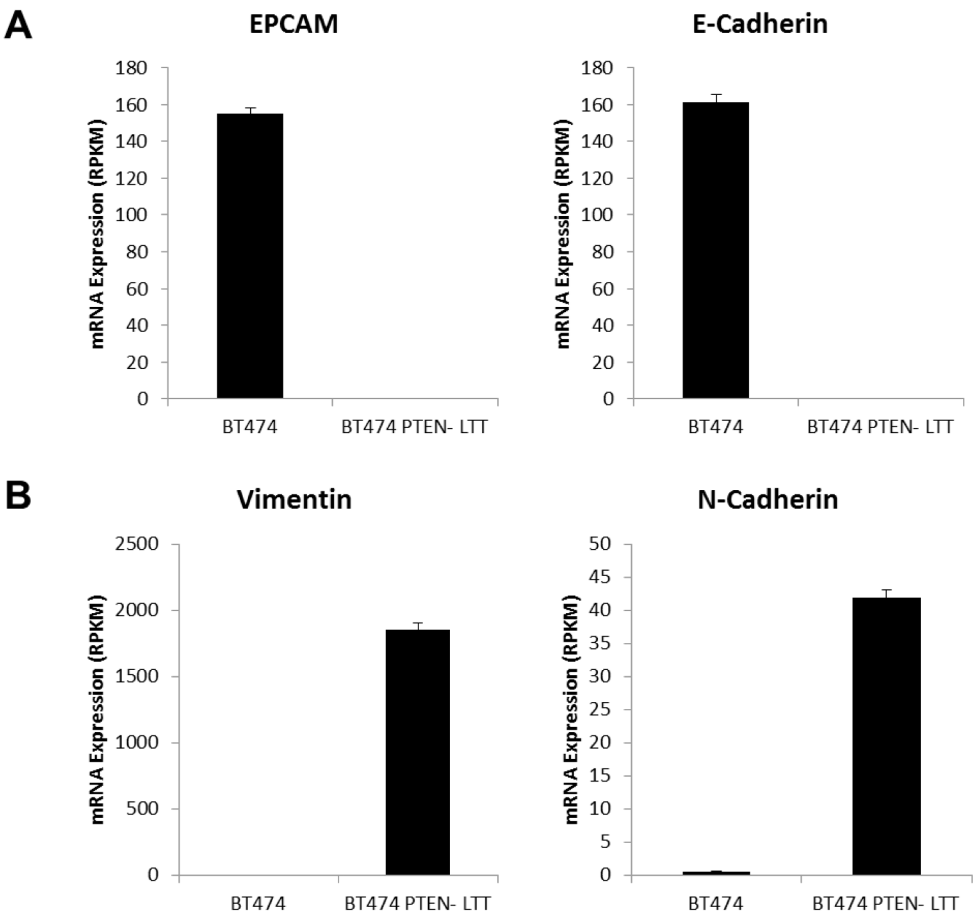

Supplementary Figure S1: Trastuzumab resistance mediated by PTEN knockdown and long term treatment induces EMT. mRNA expression of **A.** epithelial and **B.** mesenchymal markers in BT474 and BT474 PTEN- LTT cells expressed as reads/kilobase/million mapped reads as determined by RNA sequencing. N=4. Data shown as average ± SD. $p \leq 0.01$ for all genes.

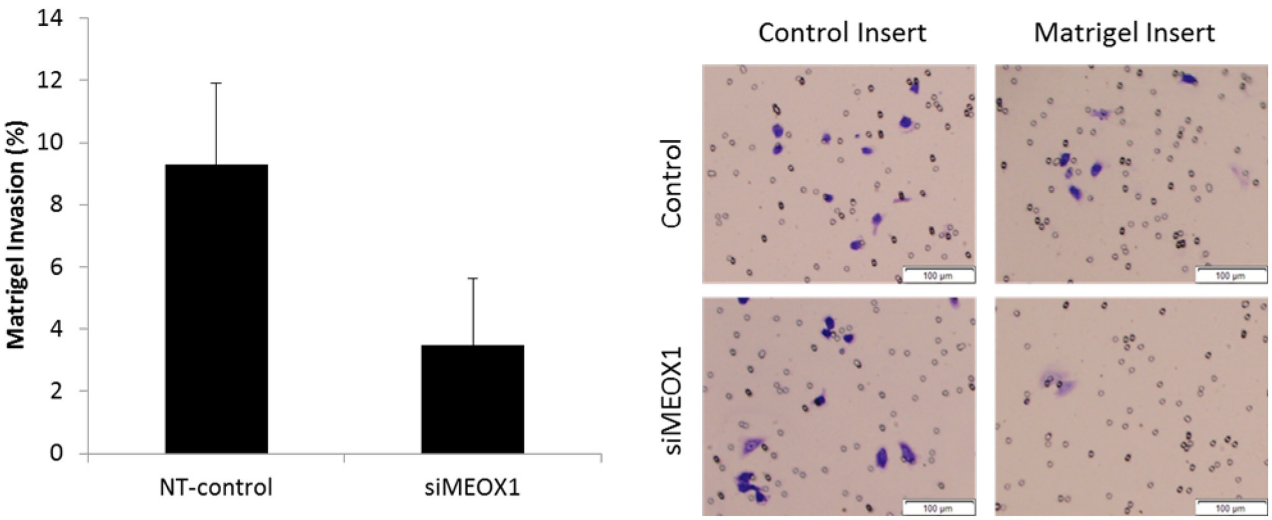

Supplementary Figure S2: MEOX1 knockdown suppresses matrigel invasion in vitro. A. Percent of BT474 PTEN- LTT cells invading into matrigel invasion chamber, normalized to control inserts. N=2, 8 independent fields of view quantified per replicate. $p \leq 0.01$. **B.** Representative image of crystal violet stained cells invaded into matrigel membrane. Scale bar = 100 µm.

Supplementary Table S1: Functional evaluation of Top 5 candidates genes toward identifying regulators of bulk proliferation and BCSC characteristics in trastuzumab resistant BT474 PTEN- LTT cells

Gene	Function	Evaluation criteria	Exclusion criteria
**CPA4**	Carboxypeptidase	Real-time PCR<br>siRNA knockdown:<br>Proliferation, mammosphere formation, colony formation, matrigel invasion	Matrigel invasion
**IGFBP3**	Growth factor binding	Real-time PCR<br>siRNA knockdown:<br>Proliferation, mammosphere formation	Proliferation assay
**LINC00-162**	Noncoding RNA	Real-time PCR<br>siRNA knockdown:<br>Proliferation, mammosphere formation, colony formation	Proliferation assay
**MEOX1**	Transcription Factor	Real-time PCR<br>siRNA knockdown:<br>Proliferation, mammosphere formation, colony formation	None
**TSHZ2**	Transcription Factor	Real-time PCR<br>siRNA knockdown:<br>Proliferation, mammosphere formation	Proliferation assay, mammosphere formation
